# Mutations in the palm domain disrupt modulation of acid-sensing ion channel 1a currents by neuropeptides

**DOI:** 10.1038/s41598-018-37426-5

**Published:** 2019-02-22

**Authors:** Benoîte Bargeton, Justyna Iwaszkiewicz, Gaetano Bonifacio, Sophie Roy, Vincent Zoete, Stephan Kellenberger

**Affiliations:** 1 Department of Pharmacology and Toxicology, University of Lausanne, 1011 Lausanne, Switzerland; 2 Molecular Modeling Group, Swiss Institute of Bioinformatics, 1015 Lausanne, Switzerland; 3 Department of Fundamental Oncology, Lausanne University, Ludwig Institute for Cancer Research, Route de la Corniche 9A, 1066 Epalinges, Switzerland

## Abstract

Modulation by neuropeptides enhances several functions of acid-sensing ion channels (ASICs), such as pain sensation and acid-induced neuronal injury. The acid-induced opening of ASICs is transient, because of a rapid desensitization. Neuropeptides containing an Arg-Phe-amide motif affect ASIC desensitization and allow continuous activity of ASICs. In spite of the importance of the sustained ASIC activity during prolonged acidification, the molecular mechanisms of ASIC modulation by neuropeptides is only poorly understood. To identify the FRRFa (Phe-Arg-Arg-Phe-amide) binding site on ASIC1a, we carried out an *in silico* docking analysis and verified functionally the docking predictions. The docking experiments indicated three possible binding pockets, located (1) in the acidic pocket between the thumb, finger, β-ball and palm domains, (2) in a pocket at the bottom of the thumb domain, and (3) in the central vestibule along with the connected side cavities. Functional measurements of mutant ASIC1a confirmed the importance of residues of the lower palm, which encloses the central vestibule and its side cavities, for the FRRFa effects. The combined docking and functional experiments strongly suggest that FRRFa binds to the central vestibule and its side cavities to change ASIC desensitization.

## Introduction

Acid-sensing ion channels (ASICs) are voltage-independent Na^+^ channels that are activated by extracellular acidification^1–3^. ASICs are expressed in the central and peripheral nervous system and contribute to the expression of fear, learning, neurodegeneration after ischemic stroke and to pain sensation^1,2,4^. The subunits ASIC1a, -1b, -2a, -2b and 3 can assemble into functional homo- or heterotrimeric channels whose biophysical properties such as pH dependence and current kinetics depend on their subunit composition^1,2^. ASICs were shown to be involved in processes associated with sustained acidification, such as inflammatory pain sensation and ischemic neuronal damage^1,5,6^. These roles require a sustained channel activity upon continuous acidification. However, extracellular acidification activates ASICs only transiently, because these channels enter rapidly a desensitized, non-conducting state. A small sustained current component is only observed in ASIC3 and in some heteromeric ASICs.

ASICs belong to the epithelial Na^+^ channel (ENaC)/degenerin ion channel family, whose members form Na^+^-permeable ion channels that are inhibited by amiloride^2^. The ENaC/degenerin family member FaNaC is a molluscan ion channel that is activated by the neuropeptide Phe-Met-Arg-Phe-amide (FMRFa)^7^. FaNaC-like channels have been described in mollusks and *Hydra*^8–10^, but not in mammals. Mammals express FMRFa-related peptides that exert their pharmacological actions mainly by binding to G protein-coupled receptors^11^. Mammalian FMRFa-like peptides contain more than four residues. Since they share the characteristic C-terminus motive Arg-Phe-NH_2_, they are also called “RFamide (RFa) peptides”^12^. ASIC modulation by RFa peptides has been documented in different heterologous expression systems, in neurons and in animal studies^7,13–15^. FMRFa and related peptides slow the desensitization time course of ASIC1- and ASIC3-containing channels and induce a sustained (non-desensitizing) current as well as an acidic shift in the SSD pH dependence of ASIC1a. The C-terminal amide group of RFa peptides is necessary for the effects on ASICs^16,17^.

**Figure 1:**
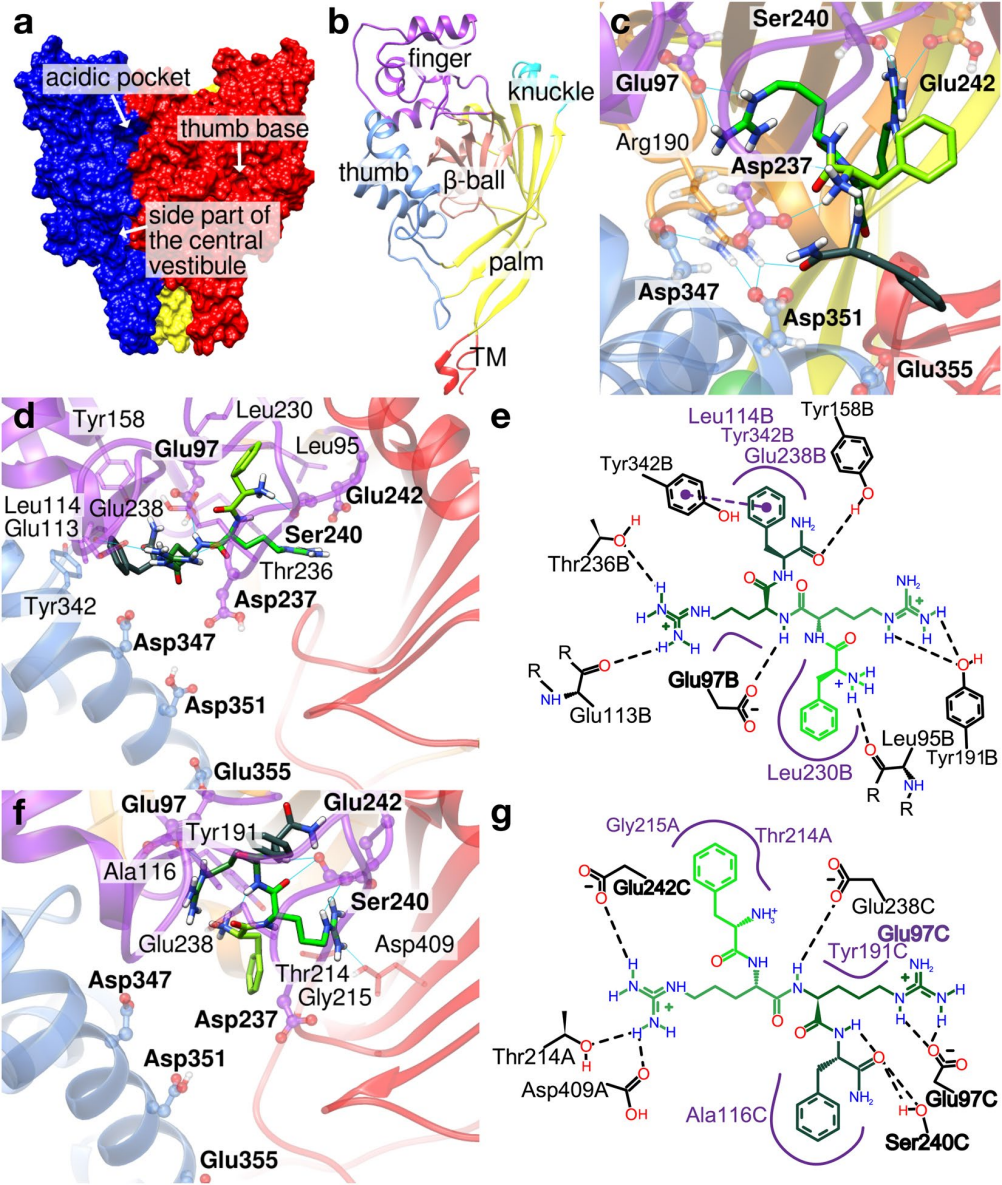
Docking of FRRFa peptide to the acidic pocket of the desensitized and closed conformation of ASIC1a. The human ASIC1a structural model is based for (**a**–**c**) on a desensitized chicken ASIC1 structure^18^, and for (**d**–**g**) on the closed chicken ASIC1^20^. (**a**) General view of the channel, highlighting the location of cavities into which peptides may bind. The central vestibule, which is enclosed by the lower palm domain, is not indicated. Estimated volumes in the desensitized ASIC1a model are ∼1600 Å^3^ (acidic pocket), ∼1150 Å^3^ (thumb base pocket) and ∼1400 Å^3^ (central vestibule, CASTp^30^). (**b**) Single subunit with structural domains indicated: transmembrane part (red), palm (yellow), β-ball (orange), knuckle (cyan), finger (purple) and thumb (blue). (**c**) Example of a predicted FRRFa interaction mode with the ASIC1a acidic pocket of the desensitized state, shown in a structural view. (**d**,**e**) One of two predicted FRRFa interaction modes with the acidic pocket of closed ASIC1a, shown in a structural view (**d**) and a schematic 2D representation (**e**) that illustrates the interactions between peptide and channel (PoseView^42^). The colours in the scheme generated by PoseView were adjusted – the peptide is coloured as in (**d** and **f**) and the hydrophobic interactions are presented in purple. The program PoseView does not apply exactly the same stringency in the detection of H-bonds as Chimera^41^, therefore there exist some differences in the H-bond attribution. (**f,g**) Example of the second predicted FRRFa interaction mode with ASIC1a in the closed conformation, in structural view (**f**) and schematic 2D representation (**g**). In (**c**,**d** and **f**) the different domains of ASIC subunits are coloured as in (**b**) for the subunit on the left, while the second subunit is shown in red. The peptide is shown in green, with light green at the N-terminus and dark green at the C-terminus; the heteroatoms of highlighted residues are colored in blue (nitrogen), red (oxygen) and white (hydrogen). Letters at the end of residue identifiers in (**e** and **g**) indicate the ASIC subunit (A, B or C). Residues that were mutated for the functional analysis are presented in ball and stick representation and labelled in bold.

Crystal structures of chicken ASIC1 show that the three subunits are arranged around the central pore. The shape of the single subunits has been compared to a forearm and a clenched hand holding a small ball. Accordingly, the different extracellular domains have been named thumb, finger, knuckle, β-ball and palm^18,19^ (Fig. 1a,b). The palm and β-ball form a β-strand-rich scaffold around the central vertical axis of the channel trimer. The lower palm domains enclose a large internal cavity in the trimer, termed “central vestibule”. The thumb and the finger form the outer, α-helix-rich channel domains, enclosing an area of high electronegativity named “acidic pocket”^18^ that collapses upon extracellular acidification^20^. Extracellular pH changes affect the protonation status of several amino acid residues located in different domains, such as the acidic pocket, the palm and the wrist, contributing thereby to activation and desensitization of the channel^18,21–25^. The palm domain undergoes large conformational changes during desensitization; its integrity is required for a normal, complete desensitization^16,22,25–27^.

Currently it is not known where in the ASICs the RFa peptides bind. Structure-function studies showed that residues in the thumb and the palm co-determine the effect of RFa peptides^16,28^, however it is not clear whether these domains participate in the formation of the binding site or whether they are important for transducing binding into functional effects. To identify potential RFa peptide binding sites on ASIC1a, we conducted *in silico* docking experiments on a human ASIC1a model with the peptide FRRFa^28^, which robustly modulates ASIC currents. Our docking studies predicted possible binding sites in the acidic pocket, a pocket at the thumb base, and in the side cavities of the central vestibule. Mutagenesis of residues being part of predicted binding sites, followed by functional analysis, demonstrated impairment of FRRFa effects in ASICs carrying mutations in the central vestibule or its side cavities, strongly suggesting this as the binding site for FRRFa.

## Results

### Molecular docking to a desensitized ASIC model predicts FRRFa binding to the acidic pocket as most probable

The *in silico* docking experiments were initially carried out with human ASIC1a homology models based on the desensitized and open chicken ASIC1 structures^18,29^, and the mutagenesis approaches in this study were based on these docking results. A crystal structure of closed ASIC, which may be more relevant in this context since RFa peptides need to be pre-applied in the pH 7.4 solution to affect ASIC current properties^15^, became available only very recently^20^. When discussing here the docking results, we present briefly the docking results on the desensitized or open ASIC1a model to illustrate the basis of the mutagenesis strategy, and then in more detail the results obtained with the ASIC model in the closed conformation.

Molecular docking was carried out with the program AutoDockVina^23^, on the trimeric ASIC1a structure comprising residues 65–432, in which the lower transmembrane and the intracellular parts were omitted (see *Methods*). The docking poses with the most favourable energy according to the internal ranking of the program were further investigated. Docking of FRRFa to the desensitized ASIC1a conformation highlighted two different cavities as most probable binding pockets, the acidic pocket and a cavity at the base of the thumb that we call here “thumb base pocket”. The docking poses in the acidic pocket (Fig. 1a) showed the most favourable docking score. The peptide docked to the acidic pocket in numerous different orientations, which were ranked closely to each other by the docking software. Since RFa peptides that contain more than four residues carry the RFa motif at the C-terminus, poses that would also be relevant for longer RFa peptides should have their N-terminal end pointing towards the outside of the pocket. Among the docking solutions whose score was within 1 kcal/mol from the best scored pose, we found several types of poses fulfilling the above criterion, one example is shown in Fig. 1c. In these poses, FRRFa interacts with several acidic residues (E97, D237, E242, E355), and other hydrophilic residues. Only sparse interaction is found for the two Phe residues of the peptide.

### Docking to the closed ASIC1a conformation shows different poses in the acidic pocket

In the closed conformation, the acidic pocket adapts an extended conformation, with the thumb α5 helix moved away from the centre of the acidic pocket, and the finger loop 229–244 shifted away from the α3 finger helix when compared with the open or desensitized conformations (Fig. 1c,d)^20^. This leads to a ∼2.3-fold increase of the volume of the acidic pocket compared to the desensitized conformation (calculated with CASTp^30^, see *Methods*) and exposes several hydrophobic residues. To select FRRFa binding positions which could also be accessible to longer RFa peptides, we carried out docking experiments with the related, but longer peptide KNFLRFa^17^. We then superimposed the complexes of ASIC1a and highest scored conformations of FRRFa (score within less than 1 kcal/mol of maximal score) with all the complexes of ASIC1a and the KNFLRFa peptide, and selected poses in which the RMSD values between the RFa moieties of both peptides was less than 1 Å. This yielded two poses in the acidic pocket (Fig. 1d–g, Supplementary Fig. S1a,b).

In contrast to the pose obtained in the desensitized state (Fig. 1c), the selected poses in the closed conformation show a peptide that does not interact with the α5-helix, but is located higher up in the acidic pocket and interacts with the finger loop, and with other parts of the finger or with the β-ball of the neighbouring subunit (Fig. 1d–g). In the first selected FRRFa pose in the acidic pocket (Fig. 1d,e), the N-terminal Phe is involved in hydrophobic interactions with Leu230 and in a hydrogen bond with the Leu95 backbone. The second Arg forms a hydrogen bond with the Tyr191 hydroxyl group, whereas Arg3 forms a hydrogen bond with the backbone of Glu113 and the side chain of Thr236. The C-terminal Phe interacts via stacking and hydrophobic interactions with Tyr342 and hydrophobic contacts with Leu114 and the hydrophobic part of the Glu328 side chain. In the second pose, the N-terminal Phe is involved in hydrophobic interactions with Gly215 and Thr214 (β-ball of neighbouring subunit), while the C-terminal Phe interacts with the side chain of Ala116 (in the finger domain). The amide function of Phe4 is involved in a hydrogen bond with Ser240 and the Arg residues and the peptide backbone form hydrogen bonds with the side chains of Glu97, Thr214, Asp409 and Glu242 (Fig. 1f,g).

### Mixed effects of acidic pocket mutations on ASIC1a modulation by FRRFa

**Figure 2:**
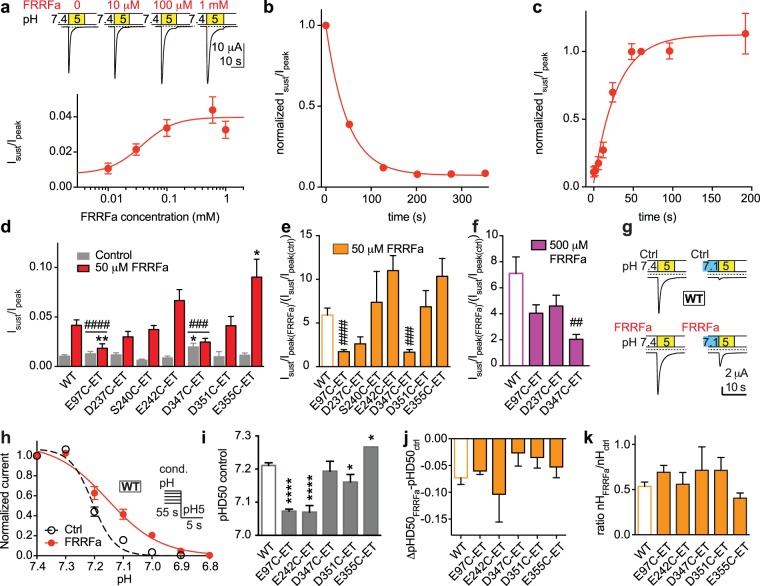
FRRFa modulation of ASIC1a currents is only partially affected by acidic pocket mutations. (**a**) FRRFa concentration-response curve. Top, representative current traces. Bottom, I_sust_/I_peak_ ratio induced by pH5 as a function of the FRRFa concentration (pre-applied during 45–50 s and present in pH5 solution), n = 7–11. (**b**) Time course of disappearance of the FRRFa-induced I_sust_. FRRFa at 50 μM was applied for 45 s, and current was measured once every minute during washout with FRRFa-free solution. Exponential fit shown as solid line (n = 4, error bars are smaller than the symbols). (**c**) Time course of appearance of I_sust_. Oocytes were exposed for various durations to 50 μM FRRFa in the pH7.4 conditioning solution, before activation of ASIC currents with pH5, including 50 μM FRRFa. Exponential fit shown as solid line, n = 4. (**d**–**f**) and (**i**–**k**), all Cys mutants had been exposed during 3 min to 1 mM MTSET before the experiment. (**d**) Bar graph of the I_sust_/I_peak_ ratio induced by pH5. Grey bars, control; red bars, after exposure to 50 μM FRRFa, n = 4–40. *p < 0.05, **p < 0.01, different from the same condition in WT; ^###^p < 0.001, ^####^p < 0.0001, the FRRFa-induced increase in I_sust_/I_peak_ (see **e**,**f**) is different from this ratio in WT. (**e**) ratio of FRRFa-induced I_sust_/I_peak_ increase by 50 μM FRRFa (n = 4–40). (**f**) ratio of FRRFa-induced I_sust_/I_peak_ increase by 500 μM FRRFa (n = 5–11). (**g**) Representative current traces of WT ASIC1a obtained at the indicated pH conditions, without FRRFa (top), with 15 μM FRRFa in the conditioning solution (bottom). (**h**) pH dependence of steady-state desensitization (SSD). Normalized pH5-induced current amplitude plotted against the conditioning pH (n = 8). The lines represent fits to the Hill equation described in *Methods*, pH protocol illustrated in inset. FRRFa was administered during the conditioning period (55 s). (**i**) Midpoint of SSD curve (pHD_50_) values obtained in the absence of FRRFa (n = 4–12). *p < 0.05; ****p < 0.0001; different from WT. (**j**) Difference in pHD_50_ (pHD_50FRRFa_-pHD_50ctrl_), n = 4–12. (**k**) nH_FRRFa_/nH_ctrl_ ratio. The pHD_50_ shifts and nH ratios of mutant channels were not different from those of WT.

ASIC1a function was measured by two-electrode voltage-clamp after recombinant expression in *Xenopus* oocytes. The channels were activated by a pH5 stimulation solution from a conditioning solution of pH7.4. FRRFa, present in both the pH7.4 and pH5 solution, induced in ASIC1a a sustained current in a concentration-dependent manner, with an EC_50_ of 37 ± 15 μM (Fig. 2a, n = 7–12). We measured the rate of FRRFa unbinding, as the disappearance of the sustained current after a 50-s incubation with 50 μM FRRFa, as 0.025 ± 0.005 s^−1^ (n = 8, Fig. 2b). The related peptide FMRFa was shown to affect ASIC1a currents only when pre-applied with the conditioning solution at pH 7.4^15^. To determine the kinetics of FRRFa association on ASIC1a, we pre-applied the peptide at a concentration of 50 μM for different time periods in the conditioning solution and measured the sustained current fraction. These experiments showed kinetics with an association rate constant k_on_ (*Methods*) of 162 ± 84 s^−1^ M^−1^ (n = 4, Fig. 2c). This shows first that the pre-application of FRRFa is necessary for the induction of a sustained current, since no sustained current increase was measured if FRRFa was only present in the pH5 solution (time point “0” in Fig. 2c), and second, that the association of FRRFa with its binding site is slow.

As mentioned above, the choice of mutations was based on the docking to the desensitized state. We mutated acidic residues at the pocket entry that may attract the positively charged peptide and control the access to the acidic pocket (Asp347, Asp351, Glu355) and in addition the residues Glu97, Asp237, Ser240 and Glu242 that were predicted to interact with FRRFa. To be more flexible in creating side chains of different properties, these residues were mutated to Cys. The mutant channels were exposed to the positively charged sulfhydryl reagent [2-(trimethylamonium)ethyl] methanethiosulfonate (MTSET, 1 mM for 3 min) before testing the effects of FRRFa. This modification creates a positively charged, large side chain^31^ that is expected to electrostatically disturb the FRRFa binding, and provide steric hindrance at the positions of interest. ASIC currents were induced by an exposure to pH5 during 10–20 s. The exposure time was kept long enough for the current to reach a steady-state, and was longer in some mutants with slower desensitization. Between applications of the acidic solution, oocytes were kept for 45–55 s in extracellular control conditioning medium at pH7.4 with or without FRRFa. The sustained/peak current amplitude ratio (I_sust_/I_peak_) at pH5, under control conditions (grey bars) and after exposure to 50 μM FRRFa (red, Fig. 2d), and the fold change of this ratio (Fig. 2e) show that the induction of sustained currents by 50 μM FRRFa was decreased by the E97C-ET and D347C-ET mutations/modifications as compared to WT. At a tenfold higher concentration, the FRRFa-induced increase of the I_sust_/I_peak_ ratio was still significantly smaller in D347C-ET than in WT, not however in E97C-ET (Fig. 2f), indicating that E97C-ET shifts the FRRFa EC_50_ to higher values, while D347C-ET suppresses the induction of a sustained current, or strongly increases the EC_50_ of FRRFa for this effect.

Moderate acidification can desensitize ASICs without apparent opening in a process that is called steady-state desensitization (SSD). The pH dependence of the SSD can be measured by exposing ASICs to a series of conditioning solutions of increasingly acidic pH for ∼1 Min, followed each time by acidification to pH5, and plotting the pH5-induced current amplitude as a function of the conditioning pH (Fig. 2g,h). RFa peptides have been shown to shift the SSD pH dependence of ASIC1a to more acidic values^28^. The inclusion of FRRFa for 1 min at a concentration of 15 μM in the conditioning solution induced an acidic shift and lowered the steepness of the ASIC1a SSD curve (Fig. 2g,h). Several of the acidic pocket mutations/modifications shifted the midpoint of SSD, termed pHD_50_ value, with regard to WT in the absence of any peptide (Fig. 2i). The FRRFa-induced shift of the pHD_50_ (Fig. 2j) and the change in steepness of the SSD curve (represented by the Hill coefficient nH, Fig. 2k) on these mutants/modifications was however not different from that observed in WT. Taken together, this analysis shows that mutation/modification of only 2 of 7 residues in the acidic pocket was able to reduce the increase in the I_sust_/I_peak_ ratio by FRRFa, and none of the tested mutations affected the FRRFa-induced shift of the SSD curve (Table 1). We consider it unlikely that FRRFa binds into the acidic pocket, since its functional effect is not prevented by mutation to Cys and subsequent attachment of a large, positively charged molecule to each of the residues D237, S240, E242, D351 and E355.

### A possible FRRFa binding site at the thumb base

**Figure 3:**
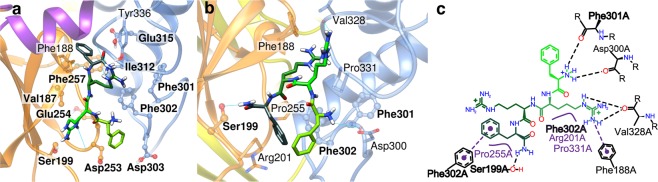
Docking results of FRRFa peptide to the thumb base pocket of the desensitized and the closed conformation of ASIC1a. The human ASIC1a structural model is based for (**a**) on a desensitized ASIC1 structure^18^, and for (**b**,**c**) on the closed ASIC1a structure^20^. (**a**) Predicted FRRFa interaction mode with the desensitized ASIC1a thumb base pocket. (**b**,**c**) Predicted FRRFa interaction mode with the closed ASIC1a thumb base pocket in structural view (**b**) and schematic 2D representation (**c**). The color code is as described in the legend to Fig. 1. Residues that were mutated for the functional analysis are shown in ball and stick representation in panels a and b and labelled in bold in all three panels.

As the docking results in the acidic pocket were not validated with the functional experiments, we further investigated the binding predictions to the desensitized channel that had obtained lower scores. In these poses, the peptide is docked at the thumb base, which is situated close to the lower end of the two thumb helices and is accessible from the “back side” of the acidic pocket, as illustrated in Fig. 1a. Since the thumb base pocket is smaller than the acidic pocket, only the Phe residues were placed inside of the pocket in high scored binding modes. Using the same reasoning as for the acidic pocket docking results, that the N-terminus of the peptide should point outwards, we considered only the poses where the Phe4 and the C-terminal amide were placed inside of the pocket. In such poses, the rest of the molecule interacts with ASIC1a residues near the pocket (Fig. 3a). FRRFa shows hydrophobic interactions with Phe257, Val187 and Phe302; Arg2 and -3 of the peptide form hydrogen bonds with the backbone of several residues.

In the closed state, the thumb base pocket has a similar volume as in the desensitized state. In the only highly ranked pose for which we detected similar conformations of the RFa moieties of FRRFa and KNFLRFa, FRRFa binds to about the same binding pocket as in the desensitized state, however its orientation is different (Fig. 3b,c and Supplementary Fig. S1c). Both Phe residues point towards the entrance of the cavity, while Arg3 is buried inside the pocket, and its guanidine moiety forms π-cation interactions with Phe188. Phe4 forms stacking interactions with Phe302, and the C-terminal amide group is involved in a hydrogen bond with the side chain of Ser199. The other specific interactions are the hydrogen bonds between the Arg3 side chain and the Val328 backbone as well as between the N-terminal amino function and the backbone of Asp300 and Phe301.

### Thumb base mutations do not prevent functional effects of FRRFa

**Figure 4:**
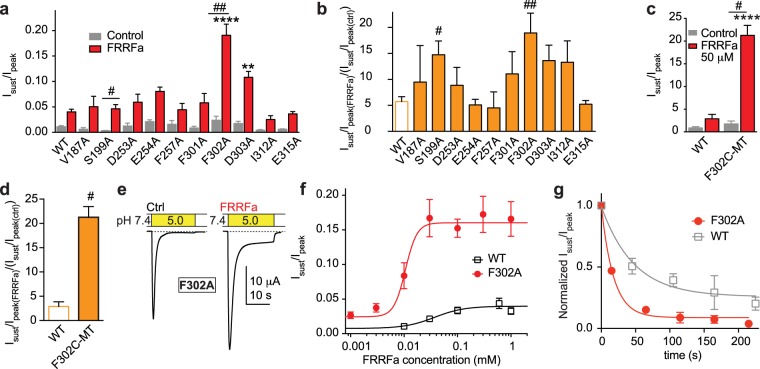
Thumb base mutations do not prevent FRRFa modulation of ASIC1a currents. (**a**) Bar graph of the I_sust_/I_peak_ ratio induced by pH5 of WT and thumb base mutants as indicated; grey bars, control; red bars, 50 μM FRRFa, n = 4–22. (**b**) Ratio of FRRFa-induced increase in I_sust_/I_peak_ in the indicated mutants (n = 4–22). For (**a**–**d**) **p < 0.01, ****p < 0.0001, different from the same condition in WT; ^#^p < 0.05, ^##^p < 0.01, the FRRFa-induced increase in I_sust_/I_peak_ is different from this ratio in WT. (**c**) Bar graph of the I_sust_/I_peak_ ratio induced by pH5 of WT F302C-MT. (**d**) Ratio of FRRFa-induced increase in I_sust_/I_peak_ in the indicated mutants. For (**c**,**d**) the oocytes had been exposed during 3 min to 1 mM MTSMT (n = 5) prior to the experiment. (**e**) Representative current traces of ASIC1a-F302A in the absence and presence of 100 μM FRRFa. (**f**) FRRFa concentration-response curve of the I_sust_/I_peak_ current ratio induced by pH5 in oocytes expressing F302A, n = 8. The WT curve is shown for comparison (n = 7–11). (**g**) Time course of disappearance of the FRRFa-induced sustained current in the F302A mutant and WT. FRRFa at 50 μM was applied for 45 s, and then washed out. The normalized I_sust_/I_peak_ ratio is plotted as a function of time. Exponential fit shown as solid line, n = 9 (F302A) and n = 4 (WT).

To scan the pocket and predicted entrance area with mutagenesis, we chose the residues with a potential to form hydrophobic (Ile312, Val187), aromatic (Phe 257, Phe301, Phe302) or electrostatic (Glu254, Asp303, Ser199, Asp253, Glu315) interactions with the peptide. For this analysis, we used mutation to Ala, and complemented the scan with mutation to Cys and subsequent chemical modification only for a selected residue. Analysis of the I_sust_/I_peak_ ratio after FRRFa showed significantly higher values than in WT for F302A and D303A (Fig. 4a). The FRRFa-induced increase in I_sust_/I_peak_ ratio was in none of the mutants significantly smaller than in WT, and was increased by the mutations S199A and F302A (Fig. 4b). Phe302 is part of a loop connecting palm and thumb domains in the close neighbourhood of the thumb base. The observed increased I_sust_/I_peak_ ratio of F302A (Fig. 4b,e) could simply be due to a better accessibility of the cavity of the putative RFa binding site in the thumb base, because Phe302 has a larger side chain than has Ala to which it was mutated. To test this hypothesis, we mutated Phe302 to Cys and exposed the mutant to the sulfhydryl reagent trimethylammonium-methyl methanethiosulfonate (MTSMT; 1 mM during 3 min) before the exposure to FRRFa. The resulting large, positively charged side chain at this position should impair FRRFa binding if Phe302 were part of the FRRFa binding site. The induction of a sustained current was however maintained in F302C-MTSMT and was similar as in F302A (Fig. 4c,d). In control experiments we confirmed that exposure of F302C to a positively charged MTS reagent changes its function, indicating thus that the Cys residue at this position is modified (Supplementary Fig. S2).

The EC_50_ of the FRRFa-mediated sustained current induction in F302A was 11 ± 2 μM (n = 8–10), compared to 37 ± 15 μM in WT (Fig. 4f). The disappearance of the sustained current followed in this mutant a faster time course than in WT (Fig. 4g, p < 0.001), suggesting that FRRFa does not have a higher binding affinity in F302A than in WT. Figure 4f illustrates that FRRFa has a higher efficacy on F302A compared to WT, as reflected by the I_sust_/I_peak_ ratio with 1 mM FRRFa of 0.03 ± 0.00 (n = 7) in WT, and 0.17 ± 0.03 in F302A (n = 9, p < 0.001). Thus, the stronger I_sust_/I_peak_ increase in F302A is mostly due to an increased efficacy. It seems therefore likely that the F302A mutation increases the effect of FRRFa by a mechanism that is independent of the binding.

### Potential FRRFa binding sites in the central vestibule

**Figure 5:**
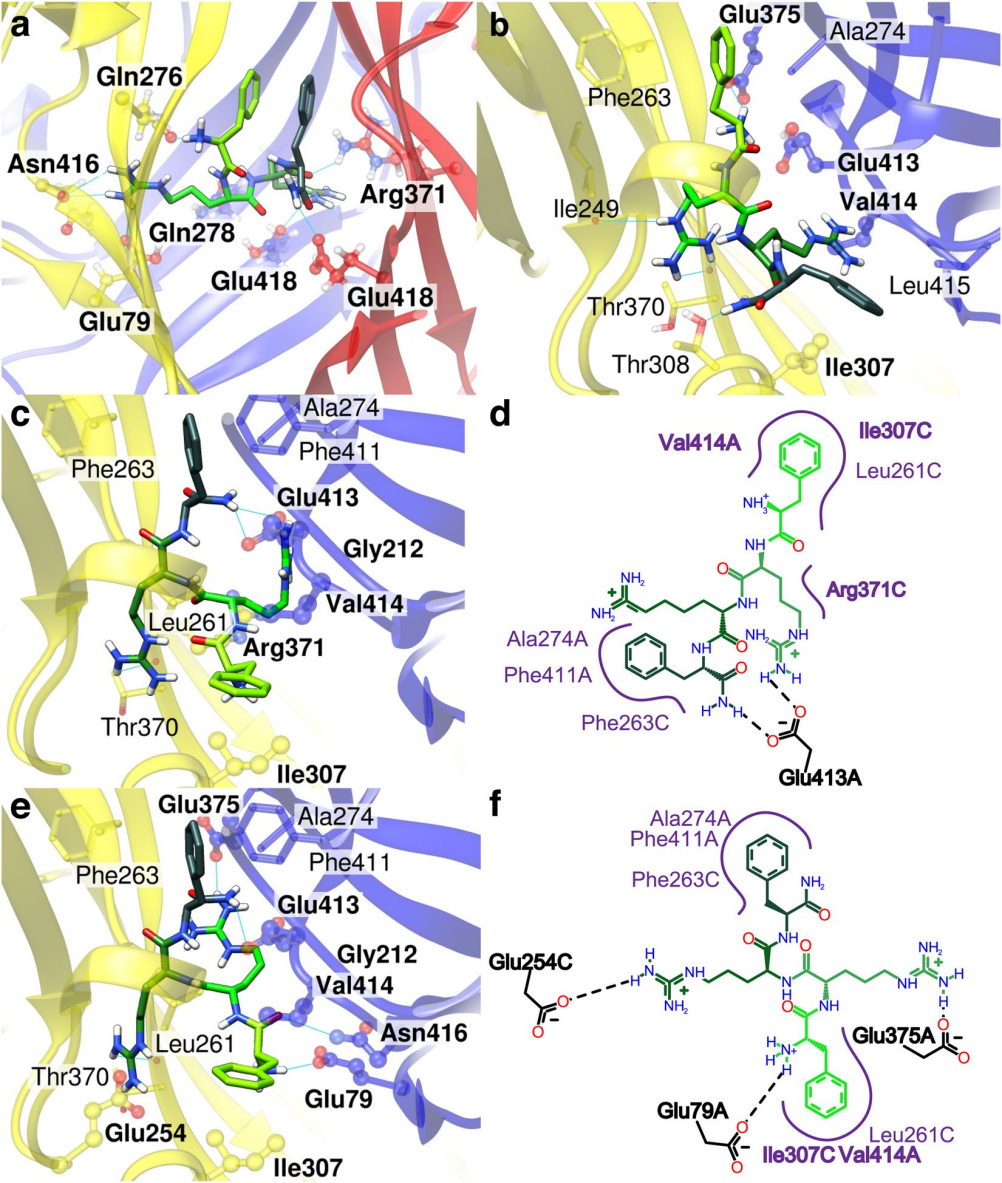
Docking results of FRRFa peptide to the central vestibule of the open and closed conformation of ASIC1a. The structural images are for (**a**,**b**) from a human ASIC1a model based on the Mit-toxin-opened ASIC1 structure^29^, and for (**c**–**f**) from a model of the closed state^20^. (**a**) Predicted FRRFa interaction mode with the central vestibule of the open ASIC1a. (**b**) Predicted FRRFa interaction mode with a side cavity of the central vestibule of the open ASIC1a. Note that the N terminus is located inside the central vestibule. (**c**–**f**) Two predicted FRRFa poses in a side cavity of the central vestibule of closed ASIC1a, in structural view (**c**,**e**) and schematic 2D representation (PoseView, modified as in Fig. 1, **d**,**f**). (**e**,**f**) For this pose, the Chimera software detected additional interactions, not detected by PoseView: a hydrogen bond between Arg3 and the Thr370 backbone and a hydrogen bond between Phe1 backbone and the Asn416 side chain. The colour code of the peptide is the same as described in the legend of Fig. 1. The three ASIC subunits are colored in yellow, red, and blue. Residues that were mutated for the functional analysis are are shown in ball and stick representation in panels a, b, c and e and labelled in bold in all panels.

To search for relevant binding positions of FRRFa, we also carried out docking experiments with a homology model based on the Mit-toxin-opened ASIC structure^29^. The conformation of most of the ASIC1 ectodomain is very similar in desensitized and open x-ray structures, however the distance between subunits is increased at the level of the lower palm and the transmembrane domains, leading to an increased volume of the cavities at these levels in the open state^26,29^. The central vestibule, located between the three palm domains^18^, is connected in the closed and open conformation to three side cavities that open between the subunits (Supplementary Fig. S3). These cavities have a total volume of ∼6500 Å^3^ in the open state, whereas the volume of the central vestibule in the desensitized state is only ∼1400 Å^3^ (CASTp). Docking poses were found with high scores in the acidic pocket and the thumb base, and with lower scores in the central vestibule. In the highest ranked poses in the central vestibule, both FRRFa Phe side chains points upwards, involved in an aromatic stacking interaction with each other (Fig. 5a). Hydrogen bond interactions are predicted with residues Glu79, Glu418, Gln276 and Asn416. In an alternative docking position, the FRRFa peptide is bound in a side opening of the central vestibule (Fig. 5b), interacting with acidic and other hydrophilic ASIC residues in the central vestibule, and with the hydrophobic amino acids Ile307, Val414 and Leu415. In this pose, the N-terminal end is however docked inside the central vestibule, thus, a longer RFa peptide could not take this pose in the same orientation.

Two binding modes of FRRFa were identified in the closed ASIC1a conformation according to the criteria of similar conformations of the RFa moiety of FRRFa and KNFLRFa (Supplementary Fig. S1d). In both poses (Fig. 5c–f), FRRFa binds into the side cavities of the central vestibule, in a similar way as previously observed from docking to the open conformation (Fig. 5b), with the difference that in the poses on the closed channel, the FRRFa N-terminus faces the outside of the side cavities. The two poses on the closed channel differ from each other in the positions of the Arg2 side chain and in the backbone of the N-terminal part, whereas the rest of the conformation is very similar. Both binding modes have the RFa moiety buried inside, with Phe4 sandwiched between Phe263 of one subunit, and Phe411 and Ala274 of the neighbouring subunit, and the amide group interacting with the Glu413 side chain. In one of the poses, Arg2 forms a hydrogen bond with the Glu413 side chain (Fig. 5c,d), whereas in the second pose the Arg2 and -3 side chains are involved in hydrogen bonds with the side chains of Glu375 and Glu254, respectively (Fig. 5e,f). In both binding modes the N-terminal Phe is placed in the hydrophobic pocket formed close to the entrance of the cavity by the side chains of Ile307, Val414 and Leu261. In the second pose, the N-terminal amino group forms a hydrogen bond with the Glu79 side chain.

To check the stability of the two poses, they were each subjected to molecular dynamics (MD) simulations of 20 ns duration (*Supplementary Methods*). Both peptides remained buried deeply in the pocket until the end of the simulation (Supplementary Fig. S4). The RMSD of the peptide backbone atoms between the docking results and structures at the end of the simulations was 1.995 Å and 2.720 Å for the poses presented in Fig. 5c–f, respectively. The phenyl ring positions were slightly changed, but the interactions with hydrophobic residues in the pocket were conserved. In both poses, the amide function and the backbone of the C-terminal RF moiety were stabilized by a rich hydrogen bond network with Glu413 and Glu375. In addition, a salt bridge between Arg3 and Glu254 was formed during the MD simulation in both systems. For the pose shown in Fig. 5c,d, Phe1 interacted with Leu369 and Val414 at the end of the simulation, and Arg2 formed hydrogen bond with Glu375, whereas in the second pose, Phe1 was located between Phe257 and Ile307, and Arg2 formed a hydrogen bond with the Asn416 backbone. Although some of the interactions of the peptide with the channel changed in the course of the MD simulations, the identified interacting amino acid residues in the central cavity are basically the same in the docking and at the end of the simulations.

### Mutations of several palm residues reduce the FRRFa-induced changes of the sustained current and SSD pH dependence

**Figure 6:**
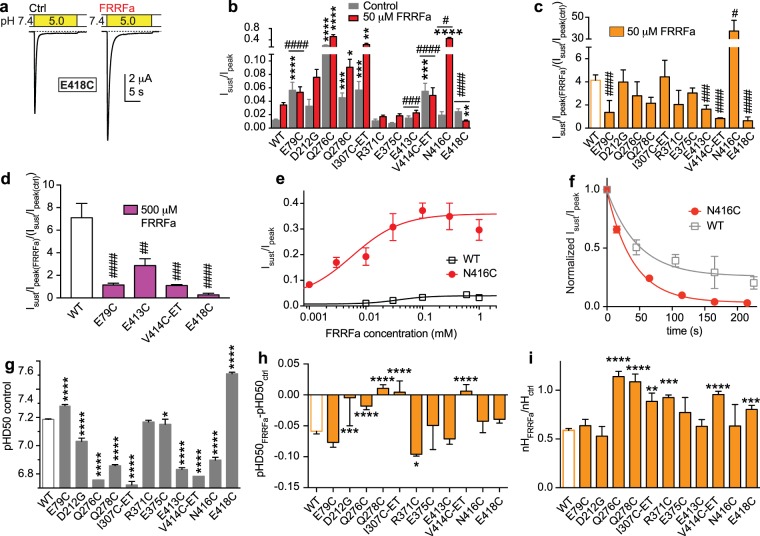
Mutations of the palm change FRRFa modulation of ASIC1a. Mutants containing “-ET” in their name had been exposed during 3 min to 1 mM MTSET prior to the experiment. (**a**) Representative current traces of the mutant E418C in the absence and presence of 50 μM FRRFa. (**b**) Bar graph of the I_sust_/I_peak_ ratio induced by pH5 of WT and mutants as indicated (n = 5–41). For (**b**,**g–i**) *p < 0.05; **p < 0.01, ***p < 0.001, ****p < 0.0001, different from WT. (**c**) Ratio of 50 μM FRRFa-induced increase in I_sust_/I_peak_ (n = 5–41). (**d**) Ratio of 500 μM FRRFa-induced increase in I_sust_/I_peak_ (n = 5–11). For (**b**–**d**) ^#^p < 0.05; ^##^p < 0.01; ^###^p < 0.001; ^####^p < 0.0001; ratio different from the ratio in WT. (**e**) FRRFa concentration-response curve of the I_sust_/I_peak_ current ratio induced by pH5 in oocytes expressing N416C, n = 8. The WT curve is shown for comparison (n = 7–11). (**f**) Time course of disappearance of the FRRFa-induced sustained current in the N416C mutant and in WT. 50 μM FRRFa was applied for 45 s, and then washed out. The normalized I_sust_/I_peak_ ratio is plotted as a function of time, and the solid line represents an exponential fit to the data, n = 9 (N416C) and n = 4 (WT). (**g**) pHD_50_ values obtained in the absence of FRRFa (n = 5–54). (**h**) The difference in pHD_50_ (pHD_50FRRFa_-pHD_50ctrl_) is plotted in the bar graph, n = 5–54. (**i**) Plot of the ratio nH_FRRFa_/nH_ctrl_ of the SSD pH dependence.

To test the docking predictions, Cys mutants of palm and other proximal residues predicted to interact with the peptide were made. Since most of these residues are large and hydrophilic, the mutation to Cys changed the side chain properties substantially. Therefore, and because MTSET modification of many palm residues leads to currents with a large sustained component^22^ that may render the measurement of FRRFa effects difficult, we exposed only the Cys mutants of hydrophobic residues (I307C and V414C) to 1 mM MTSET prior to the experiments with FRRFa. Asp212 was mutated to Gly (and not Cys), since we have previously observed that this residue is very sensitive to mutation^25^, and that it is in most ASIC1a orthologs and even in several human ASIC1a clones a Gly. Mutations of several palm residues resulted in currents with an increased I_sust_/I_peak_ ratio under control conditions (Fig. 6b). FRRFa at 50 μM induced a strong I_sust_/I_peak_ increase in the mutant N416C, and had in several mutants, E79C, E413C, V414C-ET and E418C, less effect on the I_sust_/I_peak_ ratio than in WT (Fig. 6a–c). This suggests that residues Glu79, Glu413, Val414 and Glu418 interact with FRRFa. The FRRFa-induced increase of the I_sust_/I_peak_ ratio was in these mutants smaller than that of WT also at a concentration of 500 μM (Fig. 6d). This indicates that these mutations either suppress the effect of FRRFa, or strongly increase its EC_50_. Due to the extremely small FRRFa-induced I_sust_/I_peak_ ratio changes it was not possible to determine the EC_50_ of FRRFa on these mutants. To learn more about the nature of the I_sust_/I_peak_ ratio increase by the N416C mutation, we measured the EC_50_ of FRRFa, which was 6 ± 2 μM (Fig. 6e, n = 6–7). Figure 6e also illustrates the higher efficacy of FRRFa on N416C as compared to WT ASIC1a with I_sust_/I_peak_ ratios at 1 mM FRRFa of 0.30 ± 0.04 (n = 7) for N416C and 0.03 ± 0.00 (n = 7) for WT (p < 0.0001)). Thus, the increased FRRFa-induced I_sust_/I_peak_ ratio in N416C compared to the WT is mostly due its higher efficacy on the mutant channel. The time course of the disappearance of the sustained current was not different between the N416C mutant and WT (Fig. 6f, k_off(N416C)_ = 0.027 ± 0.003 s^−1^, n = 9, compared to k_off(WT) = _0.029 ± 0.011, n = 4, in the direct comparison).

The pHD_50_ was shifted with regard to WT by many of these mutations already under control conditions (Fig. 6g). The FRRFa-induced shift in pHD_50_ was decreased in about half of the mutants, while in one of them, R371C, FRRFa induced a larger shift (Fig. 6h). Several mutations prevented the FRRFa-induced decrease of the steepness of the SSD pH dependence (Fig. 6i). Together, the effects of the mutations on the two ASIC properties (Table 1) strongly suggest an involvement of the palm in FRRFa binding.

## Discussion

Based on *in silico* docking, mutagenesis and functional validation, our study points to the side cavities of the central vestibule as the most probable binding sites for the peptide FRRFa. Previous observations resulted in hypotheses about the sites of action of RFa peptides in ASIC1a. One study showed a competition between the functional effects of PcTx1 and the peptide big dynorphin (a remotely related Arg-rich 32-amino acid peptide), which modulates ASIC1a in a similar way as RFa peptides do^32^. The 40-amino acid peptide toxin PcTx1 binds into the acidic pocket and to the subunit interface adjacent to it^26,33,34^. The observed competition may indicate a competition for the same binding site, thus that big dynorphin and possibly also RFa peptides bind to the acidic pocket or close to it. However, it also needs to be considered that PcTx1 and the neuropeptides have opposite functional effects on SSD^35,36^ and therefore, the observed antagonism may be entirely functional, with PcTx1 and big dynorphin binding to different sites on ASICs. In another study, the team of Askwith focused on the residue Leu280 of the palm. On the L280C mutant, MTSET modification strongly increases the sustained current fraction^16,22^. It was shown that 1) in the presence of FRRFa, the modification of L280C by MTSET was slowed, and 2) the FRRFa-induced increase in I_sust_/I_peak_ ratio was diminished if the channels had previously been exposed to MTSET^16^. This indicated that FRRFa interferes with conformational changes in the palm, or may impair the association of MTSET with L280C. A very recent study that applied docking and mutagenesis, suggests the central vestibule as binding site of the related peptide RPRFa on ASIC3^37^.

**Table 1:** Summary of effects of mutations on FRRFa-induced ASIC modulation.

Residue mutated*	Docking prediction^&^	I_sust_/I_peak_^§^ 50μM	SSD parameters^$^	
			ΔpHD_50_	nH_SSD_ ratio
		Compared to WT		
**Acidic pocket**				
E97	H-bond with Arg2 or Arg3	<	=	=
D237	H-bond desensitized state	=	N.D.	N.D.
S240	H-bond with Phe4 and C-terminal amide	=	N.D.	N.D.
E242	H-bond with Phe4	=	=	=
D347	Proximity, electrostatic I. desensitized state	<	=	=
D351	Proximity, electrostatic I. desensitized state	=	=	=
E355	Proximity, electrostatic I. desensitized state	=	=	=
**Thumb base cavity**				
V187	Hydrophobic I., desensitized state	=	N.D.	N.D.
S199	H-bond with C-terminal amide	>	N.D.	N.D.
D253	Hydrophobic I. desensitized state	=	N.D.	N.D.
E254	Proximity	=	=	>
F257	Stacking I. desensitized state	=	N.D.	N.D.
F301	(Backbone) H-bond with N-terminal NH_2_-group	=	N.D.	N.D.
F302	Stacking I. with Phe4	>	N.D.	N.D.
D303	Electrostatic I. desensitized state	=	=	=
I312	Hyrophobic I., desensitized state	=	=	=
E315	Electrostatic I. desensitized state	=	N.D.	N.D.
**Central vestibule and side cavities**				
E79^CV^	H-bond N-terminal NH_2_-group	<	=	=
D212^CV^	Proximity	=	<	=
Q276^CV^	H-bond open state	=	<	>
Q278^CV^	H-bond open state	=	<	>
I307^SC^	Hydrophob. I. with Phe1	=	<	>
R371^CV^	Hydrophob. I. with Arg2; H-bond open state	=	>	>
E375^CV^	H-bond Arg2	=	=	=
E413^CV^	H-bond C-term amide; H-bond Arg2	<	=	=
V414^SC^	Hydrophob. I. Phe1	<	<	>
N416^SC^	H-bond Phe1	>	=	=
E418^CV^	H-bond open state	<	=	>

*For residues of the central vestibule and its side cavities, indication of the localization, as ^CV^central vestibule; ^SC^side cavity. ^&^if not noted otherwise, docking predictions are from the closed state docking. For ^§^ and ^$^the signs indicate whether FRRFa changed this parameter in a different way in the mutant as compared to the WT; =, no significant difference. ^§^<, smaller I_sust_/I_peak_ change induced by FRRFa in the mutant with regard to WT, (>, the opposite). ^$^ΔpHD_50_, <, smaller Δ(pHD_50,FRRFa_ − pHD_50,Ctrl_) than in WT, (>, the opposite); the nH_SSD_ ratio, nH_SSD,FRRFa_/nH_SSD,ctrl_, is ∼0.5 in WT, and a higher value in a mutant than in the WT means that there is less change in the mutant; >, nH_SSD,FRRFa_/nH_SSD,ctrl_ of the mutant is higher than this ratio in WT, (<, the opposite). Electrostatic I., electrostatic interaction; Hydrophob I., hydrophobic interaction; Stacking I., stacking interaction; N.D., not determined.

Two mutations in two different regions, F302A of the thumb base pocket and N416C of the palm, boosted the FRRFa-induced increase of the sustained current. They did so mostly by increasing the efficacy of FRRFa, indicating that the mutations did not profoundly change the binding of FRRFa, but rather the consequences of FRRFa binding. While Asn416 is located close to our predicted FRRFa binding site, Phe302 is located in the thumb base pocket, for which we did not find any functional evidence as being an FRRFa binding site. Both residues are located in domains that have previously been linked to desensitization. It was shown that differences in the β9-α4 palm-thumb loop sequence – where Phe302 is located - are at the origin of differences in desensitization between mouse and human ASIC1a^28^. The palm is critically involved in desensitization^16,22,23,25,38^. Asn416 (corresponding to Asn415 in chicken ASIC1), located in the β11-β12 loop of the palm, was shown to change its side chain orientation by ∼180° between the open and desensitized state^26^. Replacement of Asn416 by positively charged side chains slows desensitization by a stabilization of the open state due to an interaction with Glu79^22^. Since Asp416 is critically involved in the conformational change during the open → desensitized transition, the N416C mutation may affect the equilibrium or the energy barrier between the open and the desensitized state, an effect that might be potentiated by FRRFa, leading to the observed increased I_sust_/I_peak_ ratio.

Our docking experiments that were the basis for the choice of mutations were carried out with models of the open and desensitized ASIC1a^19,29^. In the experimental setting, binding of FRRFa occurs mostly to closed channels, since the peptide has to be pre-applied due to its low association rate. A crystal structure of a closed ASIC has been published very recently^20^. Comparison of FRRFa docking results shows that the mutations of the thumb base pocket and the central vestibule that were tested in functional experiments cover the predicted interacting residues reasonably well. In the acidic pocket in contrast, the closed state poses were different from those obtained in the desensitized conformation. Therefore, there was less coverage by the mutagenesis approach of the interacting residues predicted from docking to the closed state. However, several tested acidic pocket residues, Glu97, Asp237, Ser240 and Glu242 are part of the FRRFa binding sites predicted from docking to the closed conformation. Since we mutated these and the other residues of the acidic pocket to Cys and modified them with MTSET that results in a positively charged adduct, these mutations/modifications should have impaired the effects of FRRFa if they depended on binding to the acidic pocket. Cys mutants at several of these positions (Asp237, Asp347, Asp351 and Glu355) were previously shown to be modified by large, Cys-reactive dyes^25^, proving the accessibility of these residues for MTSET. We show that exposure to MTSET changes the properties of the mutants E97C, S240C and E242C (Supplementary Fig. S2), indicating therefore that MTSET modifies these residues and adds a positive charge into the acidic pocket. Of the combined mutations/modifications in the acidic pocket, only two (E97C-ET and D347C-ET) prevented the FRRFa-induced increase in the I_sust_/I_peak_ ratio, and none affected the pH dependence of SSD. In contrast to the acidic pocket, 5 of 11 generally more conservative mutations in the central vestibule and its side cavities affected the FRRFa-induced increase in the I_sust_/I_peak_ ratio, and 7/11 changed the FRRFa-induced modification of the SSD (Table 1). Thus, we show here a much stronger involvement of the central vestibule and its side cavities than the acidic pocket in the effects of FRRFa. The MD simulations carried out for the peptide poses in the central vestibule of the closed ASIC1a model confirmed a stable binding of the peptide to this pocket. During the simulations, some changes of the side chain positions of Phe1 and Arg2 occurred. The position of the RF moiety changed however only slightly, and the interacting residues in the central cavity were the same in the docking and at the end of the MD simulation.

The functional analysis validated several, but not all of the interactions predicted by the docking in the central vestibule and its side cavities. A certain imprecision in the docking might be one reason for the incomplete matching of the docking and experimental findings, because it was done with a rigid homology model and did not take into account protein dynamics and flexibility that may influence the channel-peptide interactions. The MD simulations carried out with the docking poses to the central vestibule and its side cavities of the closed ASIC1a model did however not identify other interacting residues. Given the relatively low apparent affinity of FRRFa on ASIC1a, it is also expected that there are not many strong interactions between the peptide and the channel.

The reduction of FRRFa effects by ASIC1a mutations can in principle be caused by an impairment of binding, or of the steps linking FRRFa binding to changed ASIC function. The functional analysis cannot distinguish between these two possibilities. However, based on the combination of 1) the computational prediction of binding and 2) the functional effects, our data strongly suggest that the identified residues of the central vestibule and its side cavities are part of the FRRFa binding site or control its access.

## Methods

### Computational methods

The docking was carried out to human ASIC1a models of the desensitized, open and closed conformations, based on crystal structures of chicken ASIC1 that shares 90% sequence identity with human ASIC1a. For the desensitized conformation, we used a previously published structural model of human ASIC1a^23^, based on the crystal structure PDB ID 2QTS that represents a desensitized channel^18^. The human ASIC1a model of the open channel conformation was based on the PDB ID 4NTW crystal structure of chicken ASIC complexed with Mit-toxin^29^. The model of ASIC1a in the closed conformation was based on the 5WKU chicken ASIC structure^20^. Modeller software version 9.13^39^ was used to produce the homology models, in the same way as the desensitized channel model had been created^23^. In desensitized and open ASIC1a models the chloride ions were placed between the residues Arg311, Glu315 and Lys211, in analogy to the position of chloride in the chicken ASIC1 crystal structure^18^. Only the extracellular part (residues Val65 to Leu432) of the channel subunits was taken into account during the docking procedure. The protonation state of individual acidic residues was adjusted to the values at pH7.4 or pH5, according to their effective pKa values calculated previously^23^. The docking runs were performed with the channel’s acidic residues protonated according to calculated pKa values^23^ to mimic either the physiological pH7.4 (all three models) or the acidic pH5 (for the open and desensitized model). Both Arg residues of FRRFa are expected to be protonated at these pH values. Since the docking results were not different between the two protonation states, results of the docking to the desensitized and open channel are shown for a protonation state corresponding to pH5 (since these functional states occur at acidic pH), while results with the closed state model are shown for a protonation state of pH7.4. The docking was performed with AutoDock Vina^40^, version 1.1.2. The search was performed in all three subunits. The docking was repeated using each time different alpha carbon coordinates of 12 residues (Thr236, Gln278, Ile312, Lys380 from all three chains) as a search box center to ensure that the search space covered the entire extracellular part of the channel (Supplementary Fig. S5). The side length of the cubic box in which the search was performed was set to 35 Å, and the exhaustiveness parameter was set to 1000. Residues of the channel protein were kept rigid during docking.

The results of all dockings were grouped and sorted according to Vina’s internal ranking. The best poses of the ligand according to Vina were identified and their interactions with ASIC1a investigated to choose the best candidates for mutagenesis. In the case of desensitized and open channel conformations, the chosen poses fulfilled in addition the criterion of the peptide N-terminus pointing towards the entrance. In the case of closed state ASIC model docking, the filtering of the most probable poses was done using the docking results of the KNFLRFa peptide to the same channel conformation. The poses of FRRFa peptide with an energy score within a range of 1 kcal/mol from the best scored pose were compared with poses of the KNFLRFa peptide. The similarity of RFa moiety binding was measured by calculating the RMSD value over 9 heavy atoms in the peptides’ RFa groups (backbone Cα, N, side chain CZ, CG and amide NT). The conformations of FRRFa with a RMSD value lower than 1 Å were considered as similar to one of the KNFLRFa peptide and were chosen as the most relevant poses for further investigation. The docking results of FRRFa and KNFLRFa to the closed state of ASIC1a are available in the following form at the zenodo repository with the doi 10.5281/zenodo.1478585: The docking result files “FRRF.dock4” and “KNFLRF.dock4” can be opened together with the provided structural file “closed_ASIC_pH7.4.pdb” for instance with the UCSF Chimera software^41^. The pdb file “FRRF_KNFLRF_complexes.pdb“ represents the selected binding modes of FRRFa and KNFLRFa peptides in which the RF groups have similar binding positions. The docking results were visualized and analysed with UCSF Chimera. The channel-peptide interactions were represented in a schematic way using PoseView^42^. The volume of cavities was calculated with CASTp^30^ with a probe size parameter of 1.4 Å, which determines the solvent-accessible volume. It was not possible to determine the volume of the acidic pocket in the closed state with this probe size, since the acidic pocket is connected in the closed state to other cavities. To determine the fold change in volume of the acidic pocket between different functional states we used therefore a probe size of 2.0.

### Site-directed mutagenesis and expression in *Xenopus* oocytes

Human ASIC1a^43^ had been subcloned into a vector for transcription that contained 5′- and 3′-untranslated regions of *Xenopus* β-globin mRNA^44^. Point mutations were introduced using QuikChange (Agilent, Basel, Switzerland). Mutations were verified by sequencing (Synergene Biotech, Zurich, Switzerland). Expression in *Xenopus laevis* oocytes was carried out as described previously^44^. Complementary RNAs were synthesized *in vitro* (mMESSAGE mMACHINE® SP6 kit, Ambion/Life Technologies, Zug, Switzerland). Oocytes were surgically removed from the ovarian tissue of female *Xenopus laevis*, which had been anesthetized by immersion in MS-222 (2 g l^−1^; Sandoz, Basel, Switzerland). The oocytes were defolliculated, and healthy stage V and VI *Xenopus* oocytes were isolated and pressure-injected with 10 ng of cRNA or as indicated, and oocytes were kept in modified Barth saline (composed of (in mM) 85 NaCl, 1 KCl, 2.4 NaHCO_3_, 0.33 Ca(NO_3_)_2_, 0.82 MgSO_4_, 0.41 CaCl_2_, 10 HEPES, 4.08 NaOH) during the expression phase. All experiments with *Xenopus laevis* were carried out in accordance with the Swiss law on animal welfare, following protocols that had been approved by the committee on animal experimentation of the Canton de Vaud.

### Electrophysiological Analysis

Electrophysiological measurements were performed 1–2 days after cRNA injection. Ion currents were recorded using two-electrode voltage-clamp at a holding potential of −60 mV with a Dagan TEV-200 amplifier (Minneapolis, MN). Oocytes were placed in a recording chamber (300 μl) and perfused by gravity at ∼8 ml/min. Acidic (stimulation) solutions were applied once every minute for 5 s for the measurements of the pH dependence of SSD, and between stimulations the recording chamber was perfused with the conditioning solution. For the measurement of the I_sust_/I_peak_ ratio, the pH5 solution was generally applied for 10 s, and for mutants that showed a tendency to slower desensitization, for up to 20 s, to allow reaching of a steady-state sustained current. In the “FRRFa condition”, FRRFa was present in the conditioning pH solution and in the pH5 stimulation solution. When longer exposure to pH5 was chosen, the interval between the start of subsequent stimulations was increased, to expose the oocyte for at least 45 s to the conditioning solution at pH7.4 between the pH5 stimulations. The amplitude of the sustained current was measured in the last two seconds of the pH5 perfusion. Since we observed some day-to-day variation in the effects of FRRFa, we carried out on each experimental day in addition to the mutants, also experiments with WT ASIC1a. Data of mutants are presented together with the corresponding data obtained from WT at the same experimental days. The standard bath solution contained in mM, 110 NaCl, 2 CaCl_2_, 10 HEPES-NaOH (or MES- NaOH for pH < 6.8), 0.05% BSA, and the pH was adjusted with NaOH to the indicated values. The pipette solution contained 1 M KCl. Pipettes were pulled from borosilicate glass (Kimble Glass Inc., Germany). Kaleidagraph (Synergy software) or GraphPad Prism (GraphPad software, La Jolla; USA) was used to fit the pH - SSD curves to the Hill equation: I = I_max_*(1 − (1/(1 + (10^−pHD^_50_/10^−pH^)^nH^))), where I_max_ is the maximal current, pHD_50_ is the pH that induces half-maximal desensitization, and nH is the Hill coefficient. Since our setup allows switching between 8 different solutions, and two solutions are required per concentration, FRRFa concentration-response curves were constructed based on measurements made with different FRRFa concentration ranges from different cells. Concentration-response curves were fitted to a Hill-Langmuir equation. Data are presented as mean ± SEM. Differences between WT and mutant forms of ASIC1a and between different treatments were analyzed by ANOVA or Kruskal Wallis, followed by Dunnett’s or Dunn’s post hoc test, using Prism.

### Reagents

FRRFa was synthesized and lyophilized by the protein and peptide chemistry facility of the University of Lausanne or by Genescript (Hong Kong, China). Methanethiosulfonate reagents were obtained from Toronto Research Chemicals (North York, Canada), and other reagents were from Sigma, Fluka, or Applichem.

## Supplementary information

Supplementary Methods and Figures

## Data Availability

The Autodock Vina results of the docking of FRRFa and KNFLRFa peptides to the ASIC1a model in the closed conformation that can be visualised on the closed ASIC1a model, as well as the structures of selected poses of FRRFa and KNFLRFa peptides docked to the closed conformation of the ASIC1a model, are available at the Zenodo repository at https://zenodo.org/record/1478585. Other data sets are available upon request.
