# Supplementary material for: Mutations in the palm domain disrupt modulation of acid-sensing ion channel 1a currents by neuropeptides
Source: Sci Rep. 2019 Feb 22;9:2599. doi: 10.1038/s41598-018-37426-5 (PMC6385203; doi:10.1038/s41598-018-37426-5)
Supplement: Supplementary file 1 — Supplementary Methods and Figures [file 41598_2018_37426_MOESM1_ESM.pdf]

Supplementary Information to

Mutations in the palm domain disrupt modulation of acid-sensing ion channel 1a currents by neuropeptides

Benoîte Bargeton, Justyna Iwaszkiewicz, Gaetano Bonifacio, Sophie Roy, Vincent Zoete and Stephan Kellenberger

The supplementary information contains supplementary methods, five figures S1-S5, and supplementary references

Supplementary Methods: Molecular Dynamics (MD) simulations

The two complexes of the closed state ASIC1a model with the docked FRRFa peptide in the poses presented in Fig. 5c-d and Fig. 5e-f were subjected to MD simulations using GROMACS 2018.3 [1] with the CHARMM27 force field [2]. The protonation state of the protein was set to pH7.4. The complexes were solvated and the Na^+ and Cl^- ions were added to neutralize the system and to obtain 0.1 M concentration. The systems in water were subjected to 1000 steps of steepest descent minimization procedure. The cut-off value in the simulation was 12 Å and a switching function was applied on van der Waals interactions between 10 Å and 12 Å. To calculate the electrostatic interactions, the Particle Mesh Ewald method was used. The systems were heated to 300 K during 500 ps and then equilibrated during 200 ps in constant volume with position restraints of 1000 kJ/(mol nm^2) that were lowered afterwards to 300 kJ/(mol nm^2) for a 500 ps-long, constant pressure (1 Bar) simulation and finally the systems were equilibrated in constant volume during 200 ps without any position restraints. The production MD was 20 ns long in 300 K and 1 Bar pressure using the Nose-Hoover thermostat and Parinello-Rahman pressure-coupling algorithm. For the analysis, the system was superimposed with the complexes derived from docking using the receptor atoms, and the RMSD was calculated for N, CA, C and O atoms of the FRRF peptides.

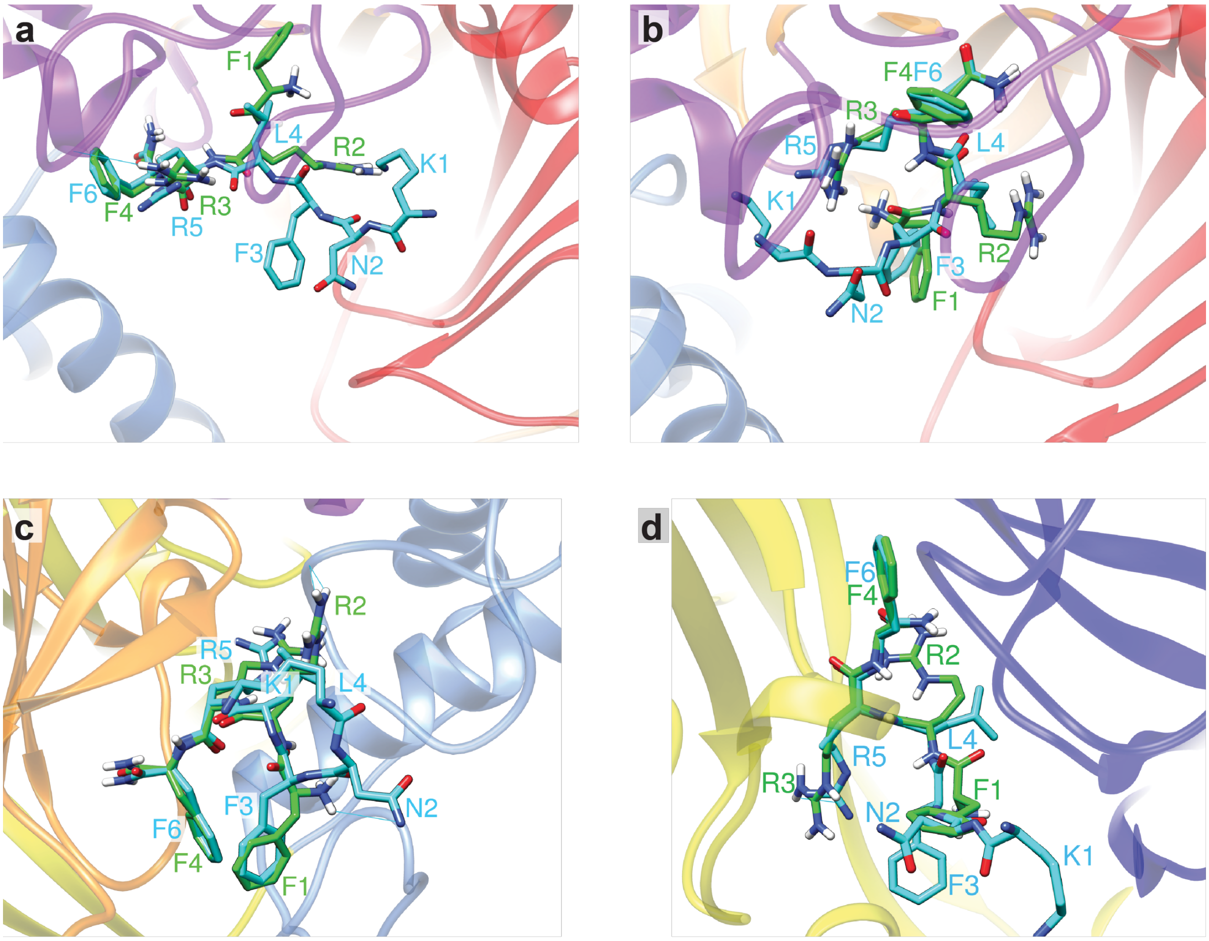

Figure S1. Superposition of FRRFa and KNFLRFa docking poses obtained with the closed ASIC1a model. a, First of the two selected poses in the acidic pocket. **b**, Second of the two selected poses in the acidic pocket. **c**, Selected pose in thumb base cavity. **d**, Selected pose in the side cavity of the central vestibule. ASIC1a domains are coloured as in the corresponding main figures, thus for **a-c**, red (transmembrane part), yellow (palm), orange (β-ball), turquoise (knuckle), purple (finger) and blue (thumb), with the neighbouring residues coloured in red in **a-b**. In **d**, the two subunits are coloured in yellow and blue. FRRFa is shown in light green, and KNFLRFa is shown in cyan. The amino acids of the two peptides are labelled in the respective colours. The heteroatoms of peptide residues are coloured in blue (nitrogen), red (oxygen) and white (hydrogen). The poses shown here fulfilled the following criteria: 1) score of the FRRFa pose within < 1 kcal/mol from the maximal Autodock Vina score, 2) RMSD between the RFa motifs of FRRFa and KNFLRFa < 1 Å (see *Methods*).

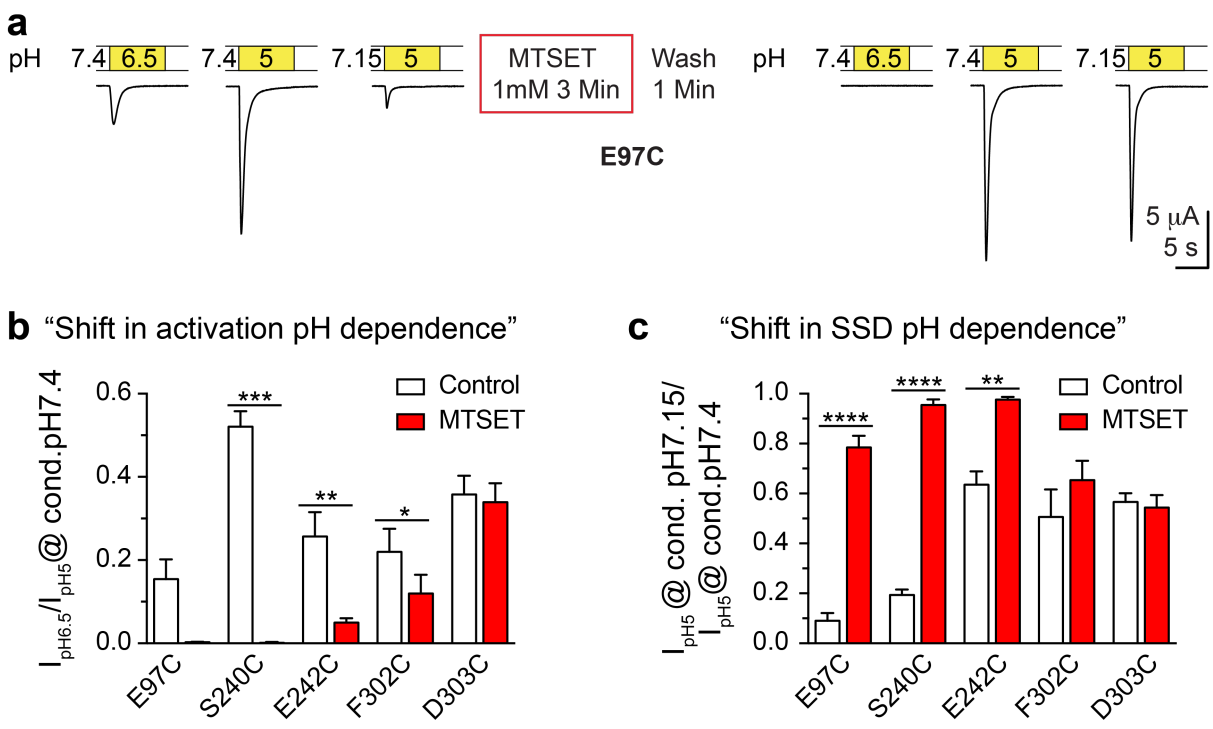

Figure S2. Modification of Cys mutants by MTSET. To determine whether exposure to MTSET modified specific Cys mutants, we applied pH protocols that detect shifts in the pH dependence of activation and of steady-state desensitization (SSD) to ASIC1a-expressing oocytes, before and after a 3-min exposure to 1mM MTSET. **a**, illustration of the experimental protocol at the example of the E97C mutant. The acidic solution was applied once every minute, except during the incubation with MTSET. **b**, ratio of current amplitudes obtained at pH6.5 and pH5 from the conditioning pH7.4, as a measure of the pH dependence of activation, before (open bars) and after MTSET exposure (red bars). **c**, ratio of the current amplitude at pH5 from the conditioning pH7.15 / the current amplitude at pH5 obtained from the conditioning pH7.4, as an indication of the pH dependence of SSD, n=4-6. *, $p<0.05$; **, $p<0.01$; ***, $p<0.001$; ****, $p<0.0001$; different between control and MTSET condition, paired t-test. We have previously shown that MTSET does not change the pH dependence of WT ASIC1a[3,4]. If a functional property is changed after MTSET incubation and its washout, it is concluded that the engineered Cys residue has been modified by MTSET. This analysis shows evidence for modification by MTSET of the mutants E97C, S240C, E242C and F302C, but not D303C.

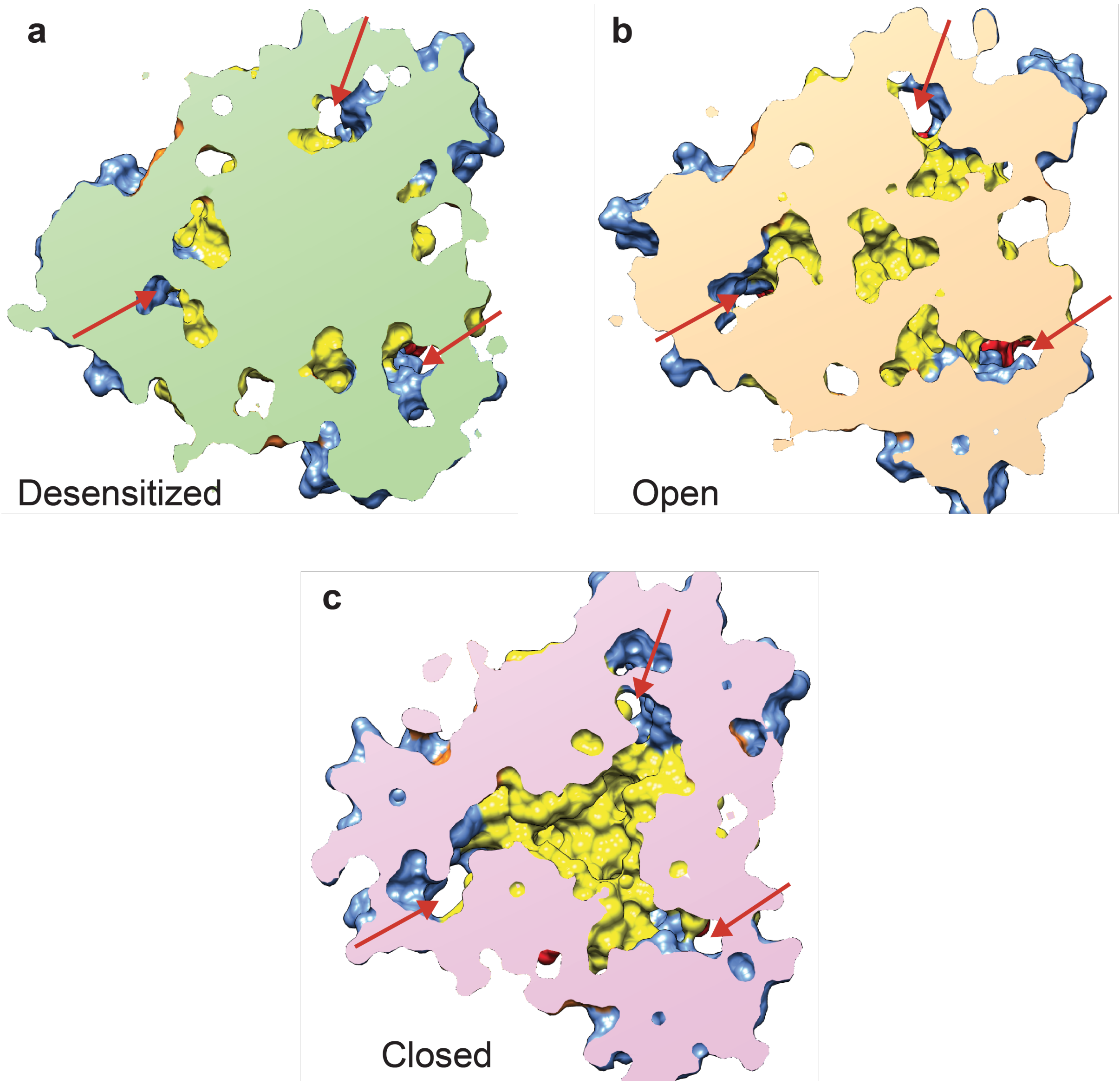

Figure S3. Structural view of the central vestibule and its side cavities. Shown is a view from top, with the channel sectioned at the level just above Asn416 and Leu415, for the desensitized (**a**), the open (**b**) and the closed channel model (**c**). The side cavities are indicated with the red arrows. The entry to the side cavities from outside is not at exactly the same level, as indicated by the white surface. Although the side cavities and the central vestibule appear not to be connected in the open state, they are, just not at the same level (**c**).

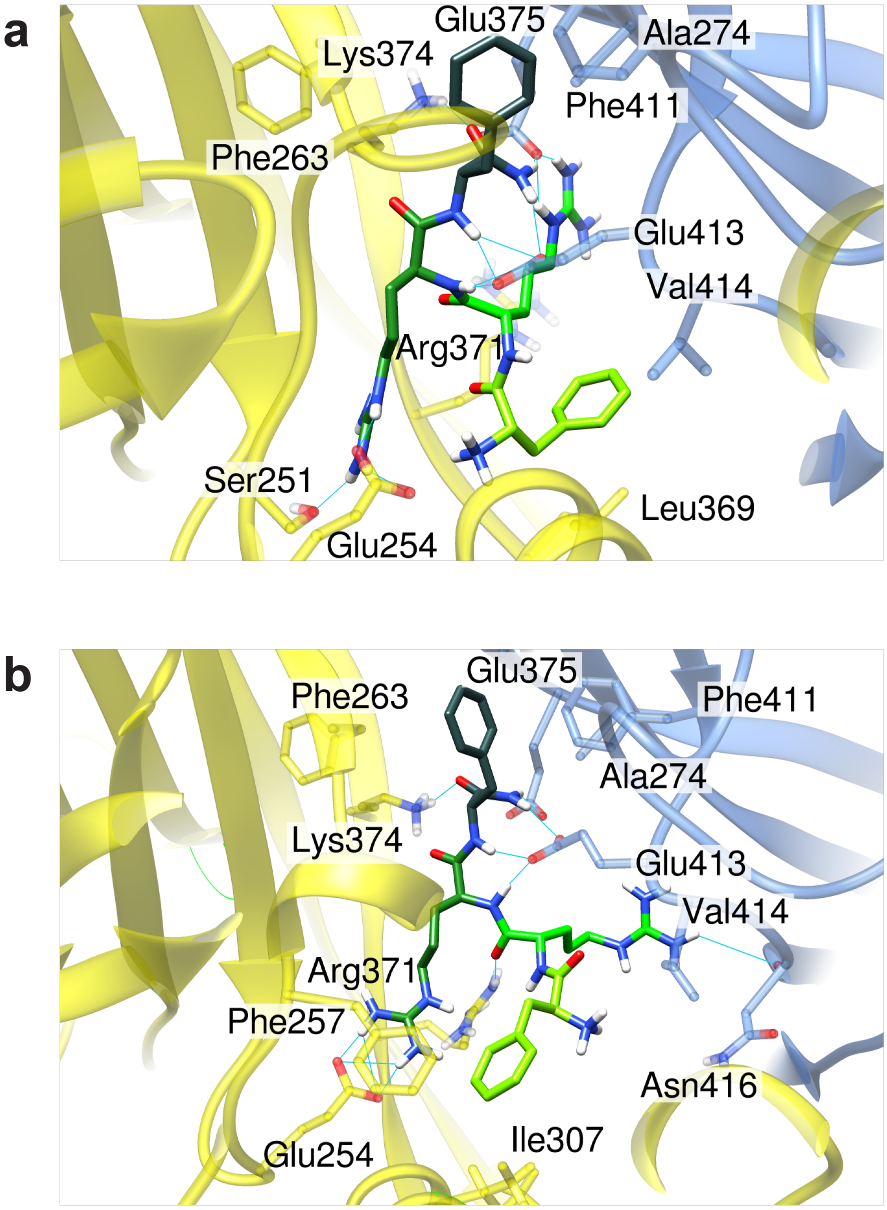

Figure S4. Structures of two binding modes of the FRRFa peptide to the closed conformation of ASIC1a after molecular dynamics simulation. a, Predicted FRRFa interaction mode with the central vestibule of the open ASIC1a, as presented in Fig.5 c-d, after 20 ns molecular dynamics in a water box. b, Predicted FRRFa interaction mode, as presented in Fig.5 e-f, after 20 ns molecular dynamics in a water box. The colour code of the peptide is the same as described in the legend of Fig. 1. The two visible ASIC subunits are coloured in yellow and blue.

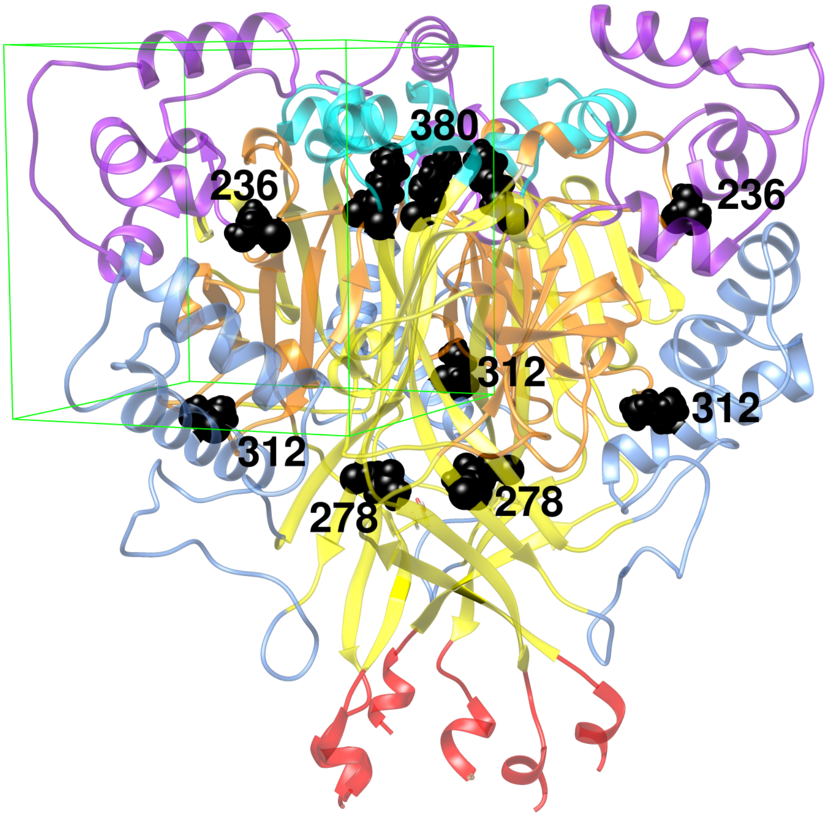

Figure S5. Docking centres used for docking of FRRFa peptide to ASIC1a. Residues whose Cα atoms were used as search box centres are presented in black and their number is indicated. As an example, the search box around residue 236 is outlined in green (see *Experimental Procedures*). ASIC1a domains are coloured in red (transmembrane part), yellow (palm), orange (β-ball), turquoise (knuckle), purple (finger) and blue (thumb).

Supplementary references

1 Berendsen, H. J. C., Vanderspoel, D. & Vandrunen, R. Gromacs - a Message-Passing Parallel Molecular-Dynamics Implementation. *Comp Phys Comm* **91**, 43-56 (1995).

2 Best, R. B. *et al.* Optimization of the additive CHARMM all-atom protein force field targeting improved sampling of the backbone phi, psi and side-chain chi(1) and chi(2) dihedral angles. *J Chemical Theory Comp* **8**, 3257-3273 (2012).

3 Roy, S. *et al.* Molecular determinants of desensitization in an ENaC/degenerin channel. *FASEB J* **27**, 5034-5045 (2013).

4 Liechti, L. A. *et al.* A combined computational and functional approach identifies new residues involved in pH-dependent gating of ASIC1a. *J Biol Chem* **285**, 16315-16329 (2010).
